# A Low-Power PLL-Less Wideband OOK Wireless Neural-Signal Transmitter for Miniaturized Neural Interfaces with In Vivo Validation in Freely Moving Mice

**DOI:** 10.3390/bios16080405

**Published:** 2026-07-25

**Authors:** Guijun Shu, Fangning Zhang, Chuang Yang, Hongyu Jia, Xiao Wang, Ming Yin

**Affiliations:** 1 State Key Laboratory of Digital Medical Engineering, School of Biomedical Engineering, Hainan University, Haikou 570100, China; shugj@hainanu.edu.cn (G.S.);; 2 Key Laboratory of Biomedical Engineering of Hainan Province, One Health Institute, Hainan University, Haikou 570100, China

**Keywords:** wireless neural recording, neural interface, OOK transmitter, PLL-less transmitter, low-power telemetry, in vivo neural recording, freely moving mice, CMOS transmitter

## Abstract

High-channel-count neural recording requires wireless links with high throughput, low power, and compact implementation, yet commercial protocols and phase-locked loop (PLL)-based transmitters often trade data rate against power and complexity. We present a low-power, PLL-less wideband on–off keying (OOK) neural-signal transmitter fabricated in a 180 nm CMOS process. The transmitter employs a free-running inductor–capacitor voltage-controlled oscillator (LC-VCO), a Gilbert mixer for OOK modulation and reverse isolation, and a current-reuse stacked power amplifier. It consumes 8 mA from a 3.3 V supply (26.4 mW), demonstrates modulation and receiver frame acquisition at a maximum raw input rate of 90 Mbps, corresponding to a 180 Mbps Manchester-coded line rate, and tunes from 3.266 to 3.445 GHz. End-to-end bit error rate (BER) was measured at raw rates of 15 and 31.2 Mbps, corresponding to encoded rates of 30 and 62.4 Mbps; the latter matches the in vivo data stream. The transmitter was integrated with a 128-channel recording chip and evaluated in freely moving adult C57 mice. Wireless hippocampal spike and local field potential (LFP) recordings, wired-system comparison, and event-locked LFP analysis support its feasibility for untethered neural recording.

## 1. Introduction

High-channel-count neural recording systems are rapidly scaling toward hundreds of channels and beyond to support the analysis of complex brain functions and behavior-related neural activity. As the number of recording channels and the sampling resolution increase, the wireless telemetry link must sustain a substantially higher data throughput. For example, a 128-channel neural recording front end with 16-bit resolution and a sampling rate of 30 kS/s per channel produces a raw neural payload rate of 61.44 Mbps. With Manchester coding, the required line rate doubles to approximately 122.88 Mbps before additional framing, synchronization, or error-checking overhead. Therefore, a low-power transmitter capable of supporting a Manchester-coded line rate at the hundred-Mbps level is important for high-channel-count wireless neural recording. Meanwhile, wireless systems for implantable or miniaturized neural interfaces are constrained by stringent power, volume, and thermal-safety requirements, and the transmitter is often one of the dominant power-consuming blocks in the wireless link. Achieving high-throughput neural data transmission within a limited power budget therefore remains a key challenge for wireless neural interface systems.

Commercial wireless protocols are not well suited to this application space. Bluetooth Low Energy (BLE) is widely adopted and power-efficient, with typical power consumption below 10 mW, but it relies on Gaussian frequency-shift keying (GFSK) over narrow channels [1,2,3,4]. This inherently limits the data rate to approximately 2 Mbps, which is insufficient for transmitting raw neural data from high-channel-count recording systems. High-speed standards such as Wi-Fi (IEEE 802.11 [5]) and 5G can readily support data rates above 50 Mbps [6,7,8]. However, their complex modulation schemes, such as orthogonal frequency-division multiplexing (OFDM) and quadrature amplitude modulation (QAM), and high-power radio-frequency (RF) front ends commonly lead to system-level power consumption in the hundreds of milliwatts to watt range. Such heat dissipation can exceed the thermal constraints of implantable neural interfaces and may increase the risk of tissue damage. Wireless power delivery may also be incorporated into the telemetry architecture. Xu et al. reported a leadless endovascular electrocorticography (ECoG) interface that combines optical data transmission with focused-ultrasound power transfer, providing an example of a jointly designed power-and-data link for implantable neural systems [9].

Custom application-specific integrated circuits (ASICs) have been developed to meet the data rate and power requirements of wireless neural telemetry. Carrier-based transmitters using amplitude-shift keying (ASK) [10,11], frequency-shift keying (FSK) [12,13], quadrature phase-shift keying (QPSK) [14], or on–off keying (OOK) often employ a phase-locked loop (PLL) for carrier synthesis [8,9,10,11,12]. For example, the Wireless Implantable Neural Recording and Stimulation system (WINeRS-8) used a PLL-generated 433 MHz carrier for a 9 Mbps OOK uplink and was validated in freely behaving rats [15]. Although PLL-based synthesis provides a stable carrier, its divider, phase-frequency detector, and charge pump add power consumption and silicon area. Impulse-radio ultra-wideband (IR-UWB) offers another approach to high-rate telemetry by transmitting short pulses or carrier bursts rather than a continuous carrier [16,17,18,19]. Recent implementations have demonstrated compact IR-UWB transmitter cores; Koleibi et al. reported a 28 nm carrier-based IR-UWB transmitter for on-cortex neural implants with a circuit area of 0.043 mm^2^ [20]. Practical IR-UWB design involves trade-offs in pulse generation and spectral shaping, wideband receiver requirements, spectral-mask compliance, and link budget. These considerations continue to motivate high-throughput transmitter architectures with reduced frequency-synthesis overhead.

**Figure 1:**
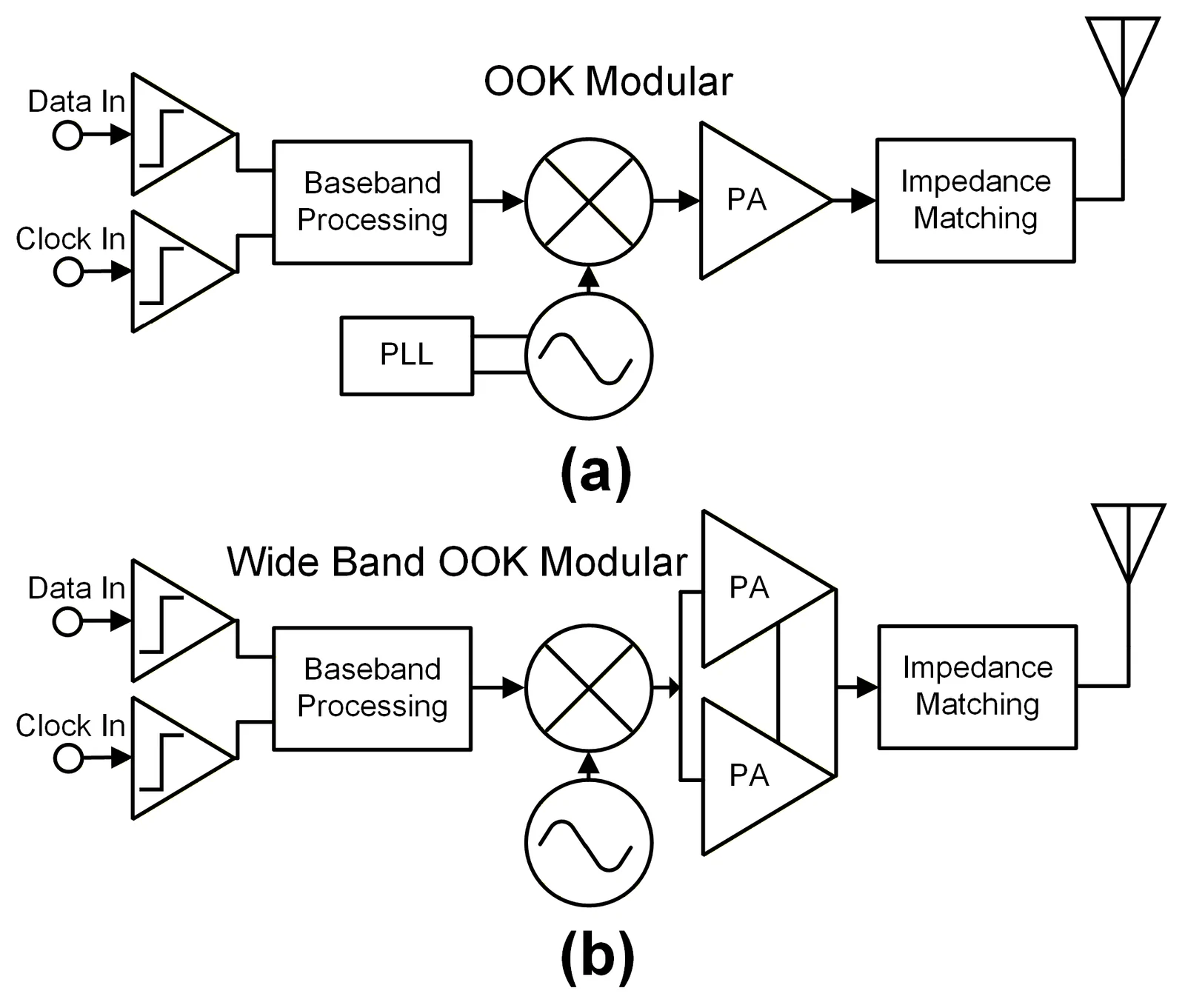
(**a**) Conventional wireless neural telemetry architecture; (**b**) Proposed PLL-less wideband OOK architecture.

Motivated by these requirements, this work presents a PLL-less wideband OOK transmitter for miniaturized wireless neural recording systems, as illustrated in Figure 1b. The transmitter uses a free-running inductor–capacitor voltage-controlled oscillator (LC-VCO) and exploits the tolerance of wideband OOK modulation to carrier-frequency offsets [21,22,23,24,25], thereby avoiding a conventional PLL-based carrier-synthesis loop [26,27]. A Gilbert mixer performs OOK modulation and provides reverse isolation between the VCO and the power amplifier (PA), while a current-reuse stacked PA improves impedance matching between the low-power output stage and the antenna. The main contributions of this work are as follows:

1. A PLL-less wideband OOK transmitter architecture is proposed for high-speed wireless neural data transmission.
2. The transmitter is implemented in a 180 nm CMOS process and supports a maximum raw input data rate of 90 Mbps, corresponding to a 180 Mbps Manchester-coded line rate, while consuming 26.4 mW. End-to-end BER is characterized at raw input rates up to 31.2 Mbps, corresponding to a 62.4 Mbps encoded line rate and matching the aggregate data rate used in the in vivo recording system.
3. The transmitter provides a tunable carrier-frequency range of 3.266–3.445 GHz, offering a hardware basis for future frequency planning in multi-node systems.
4. The transmitter is integrated with a 128-channel neural recording chip and validated through wireless neural recording in freely moving mice during T-maze exploration.

## 2. Materials and Methods

### 2.1. System Architecture

**Figure 2:**
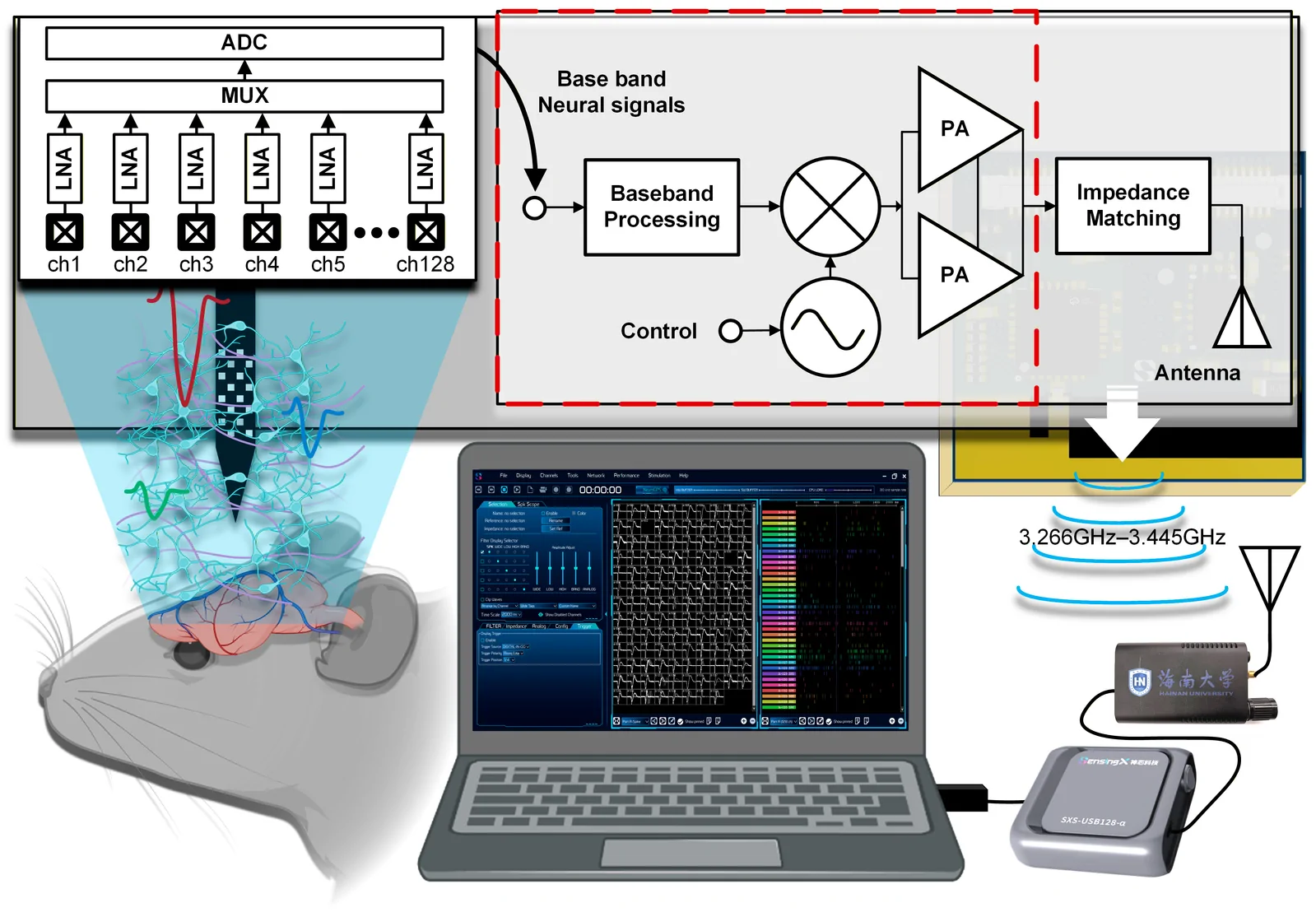
System-level block diagram of the wireless neural-signal transmission system and the proposed transmitter.

Figure 2 shows the system-level block diagram of the proposed wireless neural-signal transmitter and its integration with a neural recording front end. Neural signals are first acquired by a microelectrode array implanted in the target brain region. The signals are then amplified, filtered, and digitized by the neural recording front end. The digitized data stream is converted into a Manchester-coded digital stream suitable for wireless transmission and is then applied to the proposed wideband OOK transmitter. The transmitter operates over a tunable 3.266–3.445 GHz carrier-frequency range and consists of a free-running LC-VCO, a Gilbert-mixer modulation stage, a current-reuse stacked PA [28,29], and an impedance-matching network.

Unlike wireless neural recording systems based on general-purpose protocols such as Wi-Fi or BLE, the proposed architecture targets the specific combination of high line rate and low power consumption required for high-channel-count neural data telemetry. Conventional narrowband OOK, ASK, or FSK transmitters usually employ a PLL or frequency synthesizer to stabilize the carrier frequency against temperature variation, supply perturbation, and load pulling. However, the PLL and its associated dividers, phase-frequency detector, and charge pump increase both power consumption and silicon area. In contrast, this work adopts a PLL-less architecture. Wideband OOK modulation reduces the system’s dependence on high-precision carrier locking, while the Gilbert mixer provides reverse isolation intended to attenuate the effect of PA load variation on the VCO.

Unlike existing low-power approaches that aggressively duty-cycle the oscillator, often at the cost of spectral degradation and frequency chirping, this work uses a continuously running free-running VCO. This design decouples the trade-off between oscillator start-up time and spectral purity. In addition, the oscillator center frequency can be tuned using on-chip varactors, providing a hardware basis for future frequency planning in multi-node systems. It should be noted that the present study validates a single-node wireless neural recording system; simultaneous multi-node FDMA operation [30] remains to be investigated in future work.

### 2.2. Transmitter Circuit Design

**Figure 3:**
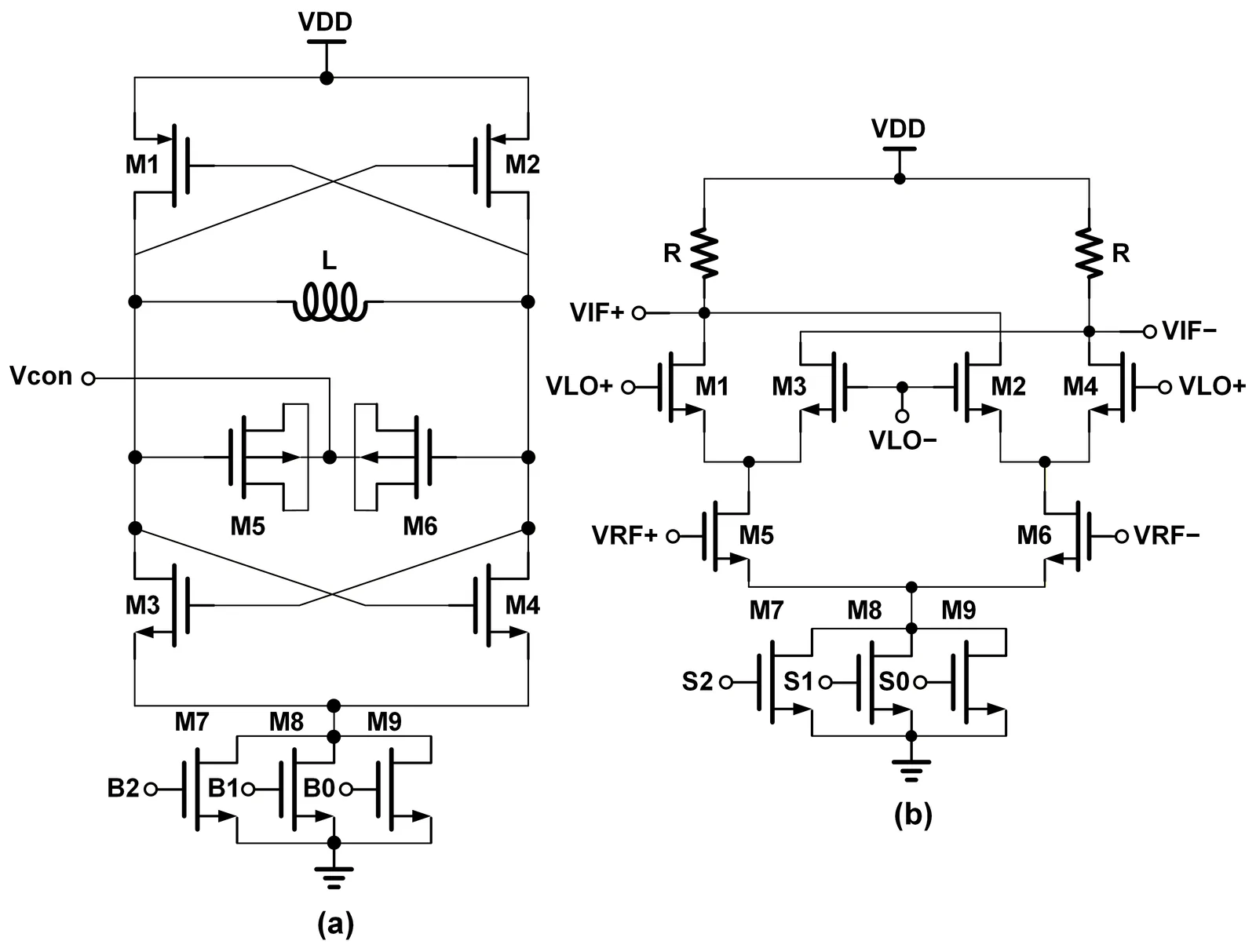
Core circuits of the wireless neural-signal transmitter: (**a**) Frequency- and gain-tunable LC-VCO. (**b**) Gain-tunable mixer.

As shown in Figure 3a, the proposed design uses a continuously running, power-tunable LC-VCO rather than a duty-cycled oscillator that repeatedly undergoes start-up transients. This topology avoids spectral broadening caused by frequency chirping during signal build-up. At an offset angular frequency Δω from the carrier $\omega_{0}$, the steady-state phase noise $L\left(∆\omega \right)$ can be approximated by the linear time-invariant Leeson model:

(1) $$L\left(∆\omega \right)=10\log\left[\frac{2FkT}{P_{sig}}\left(1+{\left(\frac{\omega_{0}}{2Q∆\omega }\right)}^{2}\right)\right]$$

Here, F is the device noise factor, k is the Boltzmann constant, T is the absolute temperature, $P_{sig}$ is the signal power, and Q is the loaded quality factor of the resonant tank. In a duty-cycled oscillator, the effective Q can be reduced during the transient phase and the center frequency may drift dynamically, $\left(\omega (t)\neq \omega_{0}\right)$ By keeping the VCO in steady-state operation, both $P_{sig}$ and Q are maximized and remain relatively stable during transmission.

To implement OOK modulation with a continuously running VCO, a power-tunable Gilbert-cell up-conversion mixer is used, as shown in Figure 3b. One key function of this stage is to provide reverse isolation and prevent load variations at the PA from perturbing the VCO frequency, a phenomenon commonly known as load pulling. The frequency pulling $∆\omega_{pull}$ caused by a load impedance variation can be approximated as:

(2) $$∆\omega_{pull}\approx \frac{\omega_{0}}{2Q}\cdot \frac{\left|\Gamma_{load}\right|}{1-{\left|\Gamma_{load}\right|}^{2}}\cdot \sin\left(\phi_{load}\right)$$

Here, $\Gamma_{load}$ denotes the reflection coefficient seen by the oscillator tank. In a direct-drive architecture, Gamma_load can vary significantly between logic “1” (PA on) and logic “0” (PA off). In the proposed design, the Gilbert mixer acts as a buffer. The reflection coefficient variation seen by the VCO is attenuated by the reverse isolation of the mixer:

(3) $$\left|\Gamma_{VCO}\right|\approx \left|\Gamma_{PA}\right|\cdot {\left|S_{12,mixer}\right|}^{2}$$

The proposed Gilbert cell uses a double-balanced mixer structure with resistive loads. The bottom transconductance stage converts the input RF voltage into a differential current, while the LO-driven switching quad periodically commutates the current to achieve frequency conversion. The top resistive loads convert the mixed current into a differential output voltage. Compared with a single-ended mixer, the double-balanced topology suppresses direct LO and RF feedthrough and improves port-to-port isolation.

In ultra-low-power transmitters, the high transistor output resistance, $R_{opt}\approx V_{swing}/I_{DC}$, can be poorly matched to a conventional 50 ohm antenna, thereby limiting the overall efficiency. To address this issue, a current-reuse stacked PA topology is adopted. The circuit consists of two amplifier stages stacked in the DC path to reuse bias current while being combined in parallel for AC signal operation. The effective output resistance is approximately the parallel combination of the two stages:

(4) $$R_{out,stacked}=R_{out,top}∥R_{out,bot}\approx \frac{1}{2}R_{out,single}$$

Similarly, the optimum load resistance becomes:

(5) $$R_{opt,stacked}\approx \frac{1}{4}\frac{V_{DD}}{I_{DC}}$$

This relationship indicates that, compared with a single transistor driven under the full supply voltage, the stacked topology naturally reduces the effective source resistance. This impedance transformation moves the PA output impedance closer to 50 ohm and can reduce insertion loss in the matching network.

To assess the intrinsic behavior of the free-running oscillator, schematic-level simulations of the LC-VCO were performed using SpectreRF. The simulations evaluated the effects of temperature, supply voltage, and resistive loading on the oscillation characteristics, together with phase-noise performance at the nominal operating point.

### 2.3. ASIC Fabrication

**Figure 4:**
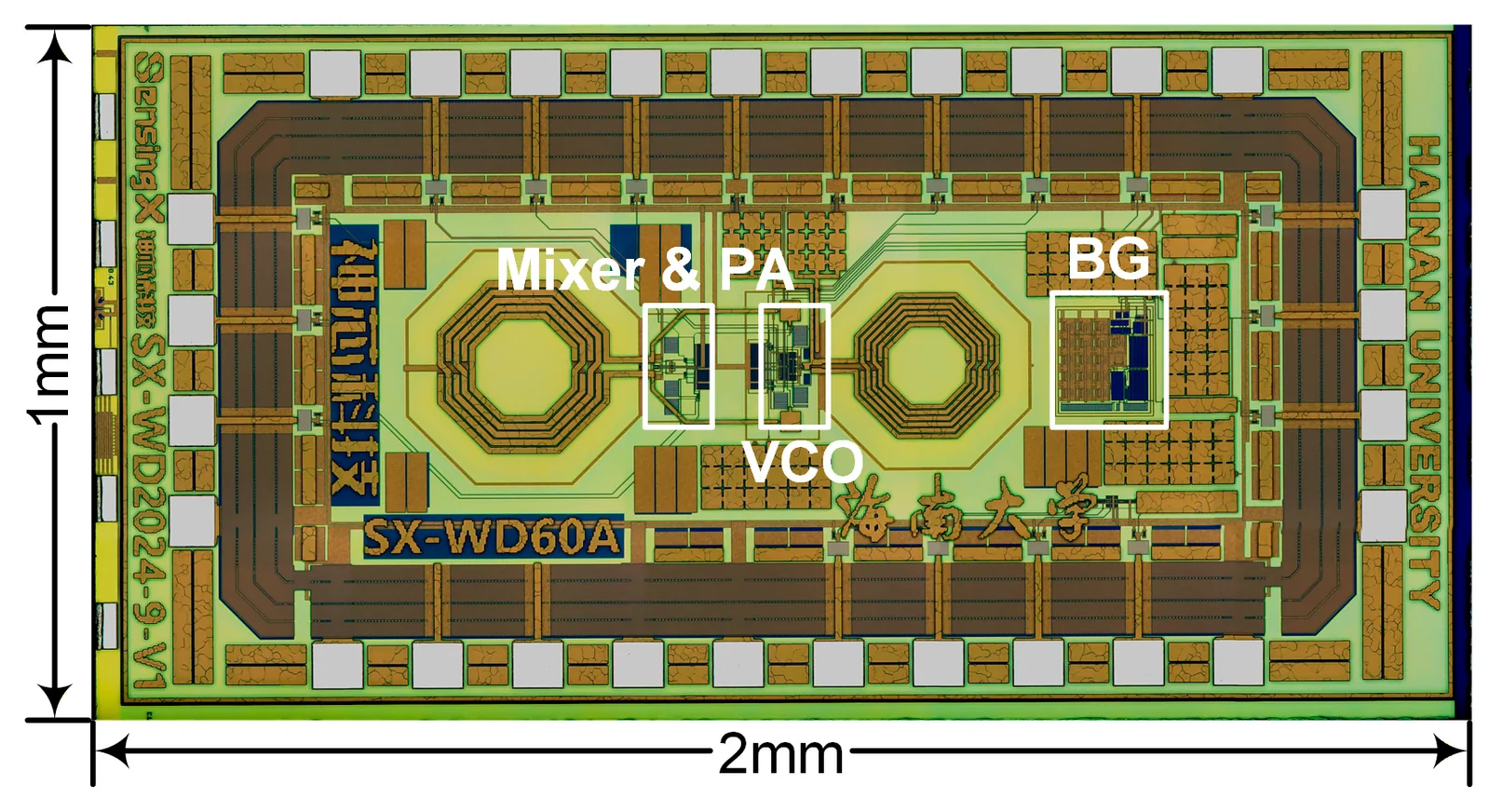
Chip micrograph of the wireless neural-signal transmitter. VCO, voltage-controlled oscillator; PA, power amplifier; BG, bandgap reference.

The proposed wireless neural-signal transmitter was implemented in a 180 nm CMOS process. The chip occupies a silicon area of 2 mm^2^, as shown in Figure 4.

### 2.4. Animal Surgery and Behavioral Experiment

Adult C57 mice were used for chronic electrode implantation. Under isoflurane anesthesia at an appropriate dose, a 32-channel microelectrode array was stereotactically implanted into the dorsal hippocampus. The ground silver wire was fixed to the skull above the cerebellum using a reference screw, and the electrode interface was secured with dental cement. All animal procedures were approved by the Institutional Animal Care and Use Committee.

After a two-week postoperative recovery period, the wireless neural recording device was connected to the head-mounted electrode interface and mounted on the back of the mouse. Before behavioral recording, the mice were habituated to the T-maze environment. During the experiment, the mice freely explored the T-maze, and their trajectories were recorded by an overhead industrial camera at 30 frames/s. When predefined behavioral events were detected, the behavioral tracking software sent 3.3 V TTL synchronization pulses to the neural recording system through a data acquisition card, enabling temporal alignment between behavioral events and neural signals.

For the wired–wireless comparison, recordings were obtained from the same animal through the same electrode interface during the same T-maze behavioral task. The wired recording was performed first, followed by wireless recording under comparable behavioral conditions. Both systems operated at a sampling rate of 15 kS/s with 16-bit analog-to-digital conversion.

### 2.5. Neural Signal Processing and Statistical Analysis

For the in vivo validation in freely moving mice, neural signals were acquired at a sampling rate of 15 kS/s with 16-bit ADC resolution. All neural-signal processing was performed using the actual sampling rate recorded in the corresponding data files. To extract spike activity, the raw signals were first median-centered and then band-pass filtered at 300–3000 Hz to obtain high-frequency spike components. Spike events were detected using a robust threshold method based on the noise level estimated from the median absolute deviation. The detection threshold was set to 4.5 times the estimated noise level, and a 1 ms refractory window was applied to avoid repeated counting of the same event. The detected spike events were used for waveform analysis, inter-spike interval (ISI) calculation, and principal component analysis (PCA) projection to evaluate the recording quality of high-frequency neural activity in the wireless data. The spike analysis in this work was used for system-level functional validation and signal-quality assessment; spike events were not assigned to single-neuron identities.

LFP signals were obtained by filtering the raw signals in the 1–250 Hz band. Event-locked LFP bandpower analysis was performed using Welch power spectral density estimation, and bandpower was computed by integrating the power spectrum over the target frequency bands. The analyzed bands included delta (1–4 Hz), theta (4–12 Hz), beta (13–30 Hz), and gamma (30–80 Hz). The event window ranged from 5 s before to 5 s after each behavioral event, with a time-frequency bin size of 0.5 s. The baseline window was defined as −5 s to −2 s relative to the event. Bandpower values were log10-transformed and baseline-z-scored.

The primary endpoint was defined a priori as the difference in baseline-z-scored log10 bandpower between the post-event window [0, 1 s] and the pre-event window [−2, −1 s]. The mouse was used as the highest independent statistical unit, whereas sessions, trials, and channels were treated as nested repeated observations. Behavior-related changes in LFP bandpower were interpreted as exploratory results for functional validation of the wireless system rather than as independent evidence for a specific neural mechanism. Data processing was performed in Python 3.11.15 using NumPy 2.4.6, SciPy 1.17.1, pandas 3.0.3, Matplotlib 3.10.9, and neo 0.14.4.

## 3. Results

### 3.1. Transmitter Implementation, RF Characterization, and BER Measurement

**Figure 5:**
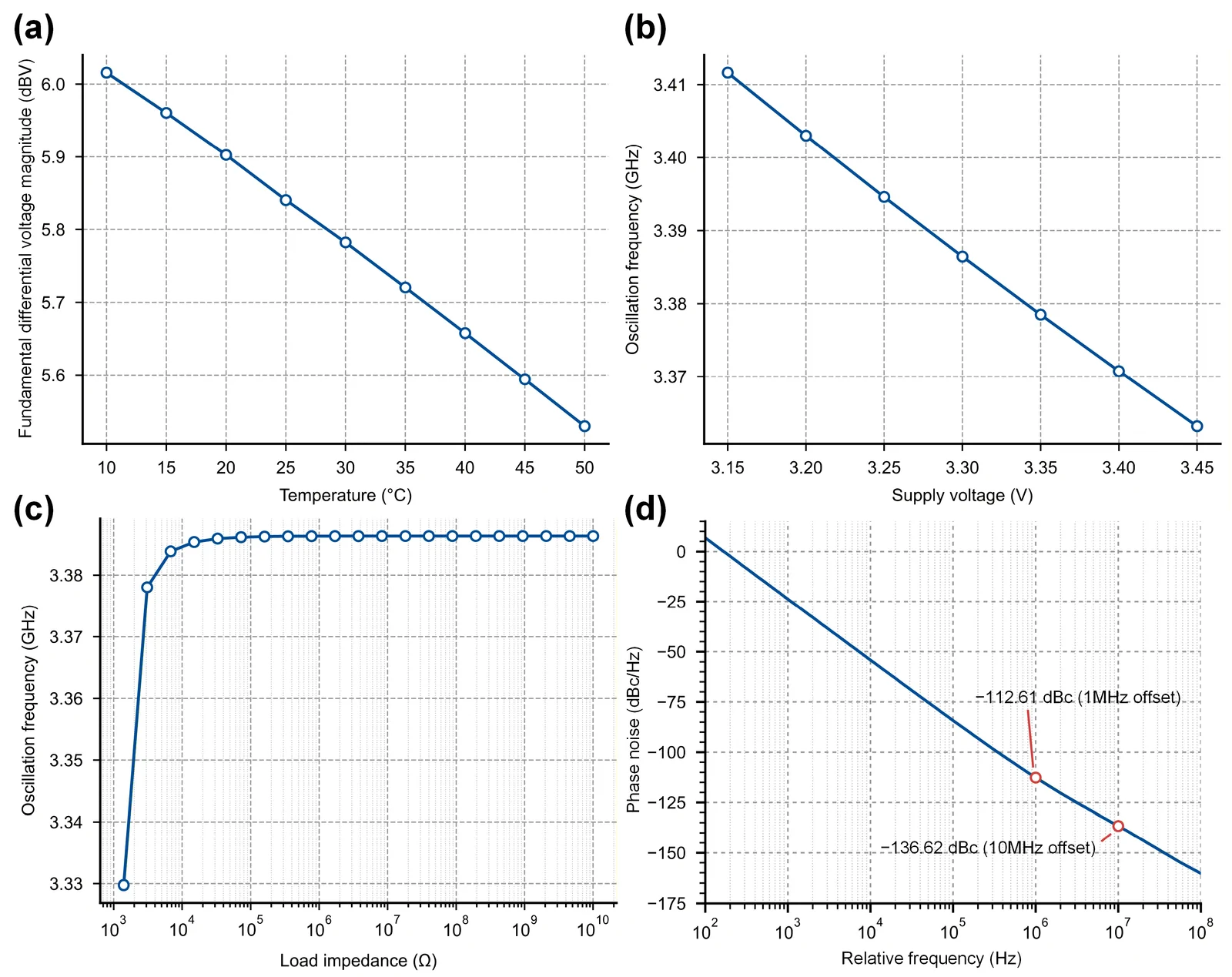
Schematic-level simulation results of the LC-VCO at the typical-typical process corner. (**a**) Simulated fundamental output component remained at approximately 3.38 GHz over a temperature range of −15 to 50 °C under a 3.3 V supply and a 1 GΩ resistive load. (**b**) Oscillation frequency versus supply voltage from 3.15 to 3.45 V at 27 °C with a 1 GΩ load. (**c**) Oscillation frequency versus resistive load from 1.4 kΩ to 10 GΩ at 27 °C and a 3.3 V supply. (**d**) Simulated phase-noise spectrum at 27 °C, 3.3 V, and a 1 GΩ load.

Figure 5 summarizes the schematic-level behavior of the LC-VCO at the typical-typical process corner. As shown in Figure 5a, the fundamental output component remained near 3.38 GHz over the simulated temperature range, while its amplitude changed only slightly. Sweeping the supply voltage from 3.15 to 3.45 V produced a frequency shift of 50 MHz, as shown in Figure 5b. This shift was small relative to the 179 MHz carrier-frequency tuning range of the transmitter. The load sweep in Figure 5c shows that reducing the resistive load from 10 kΩ to 1.4 kΩ shifted the oscillation frequency by approximately 50 MHz. Above 10 kΩ, the simulated frequency changed only slightly. This sweep represents the VCO cell under direct resistive loading and does not include the reverse isolation provided by the Gilbert mixer in the complete transmitter. It should therefore be interpreted as a cell-level sensitivity analysis rather than a direct measurement of packaged-transmitter load pulling. At the nominal operating condition, the simulated phase noise was −112.6 dBc/Hz at a 1 MHz offset and −136.6 dBc/Hz at a 10 MHz offset from the 3.38 GHz carrier, as shown in Figure 5d.

**Figure 6:**
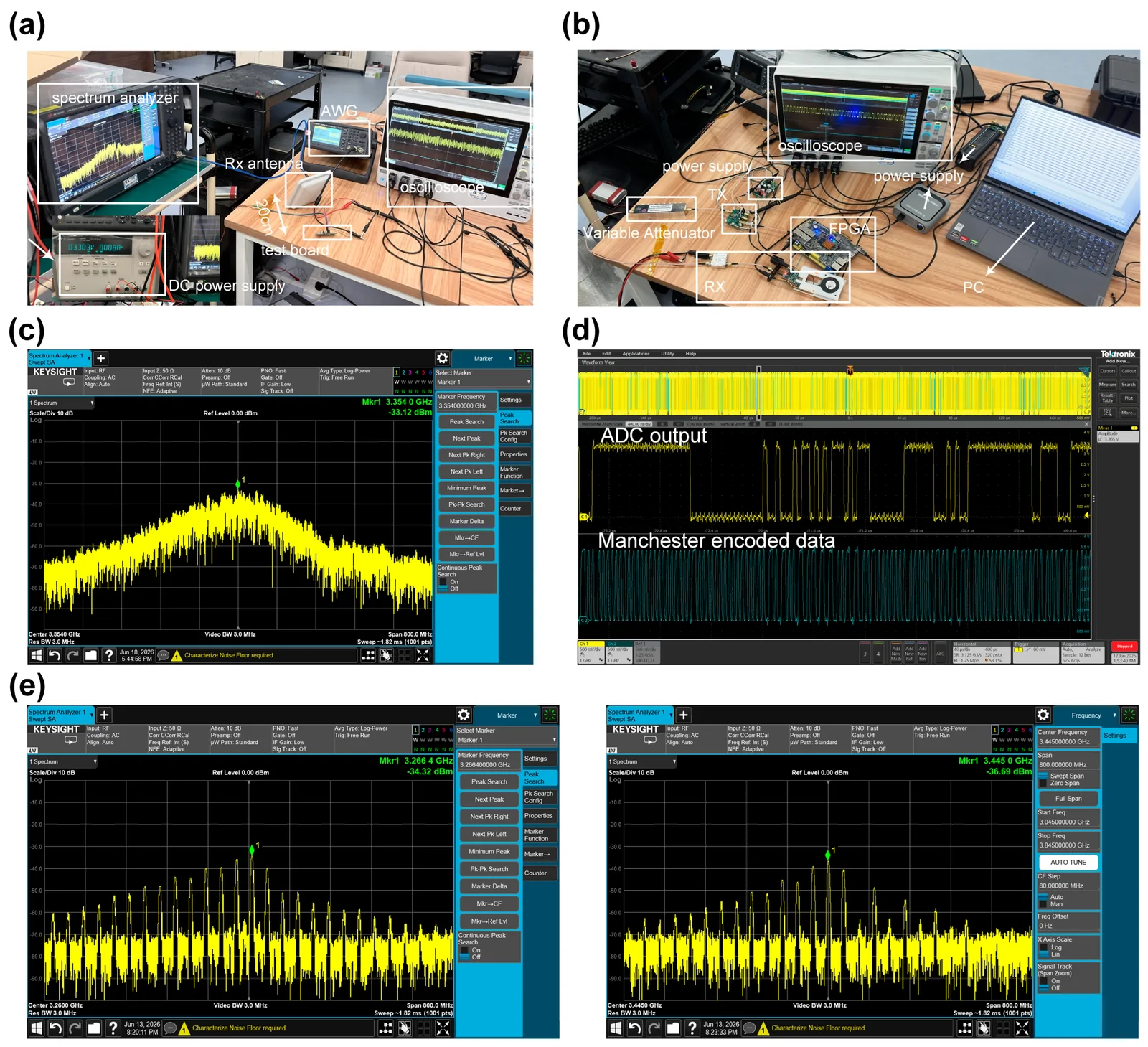
Electrical and link-characterization setups and measured transmitter outputs. (**a**) Bench-top RF measurement setup. The transmitter was powered from a 3.3 V supply and drew 8 mA. (**b**) FPGA-based end-to-end BER measurement setup, including the transmitter, variable attenuator, receiver, FPGA return path, and serial connection to the host computer. (**c**) RF spectrum of the transmitter with a 90 Mbps raw PRBS input after on-chip Manchester encoding and OOK modulation. (**d**) ADC output from the 128-channel neural recording chip and the corresponding on-chip Manchester-coded output waveform of the transmitter. (**e**) Frequency tuning of the wireless neural-signal transmitter over a 3.266–3.445 GHz range with a 179 MHz tuning bandwidth; the left and right spectra show carrier frequencies of 3.266 GHz and 3.445 GHz, respectively.

The proposed wireless neural-signal transmitter was fabricated in a 180 nm CMOS process, and the chip micrograph is shown in Figure 4. The chip occupies a silicon area of 2 mm^2^. After quad flat no-lead (QFN) packaging, the chip was mounted on a custom printed circuit board (PCB) for bench-top characterization. The RF measurement setup is shown in Figure 6a. Under a 3.3 V supply, the transmitter drew 8 mA, corresponding to a DC power consumption of 26.4 mW.

To evaluate high-speed modulation capability, a 90 Mbps raw PRBS input was applied to the transmitter and Manchester-encoded on-chip, corresponding to a 180 Mbps encoded line rate. The resulting wideband OOK spectrum was centered at 3.354 GHz, as shown in Figure 6c. The transmitter was also connected to the 128-channel neural recording chip. Figure 6d shows the ADC output from the recording chip and the corresponding Manchester-coded transmitter input waveform. By tuning the on-chip varactors, the carrier frequency was adjusted from 3.266 to 3.445 GHz, corresponding to a tuning range of 179 MHz, as shown in Figure 6e.

**Figure 7:**
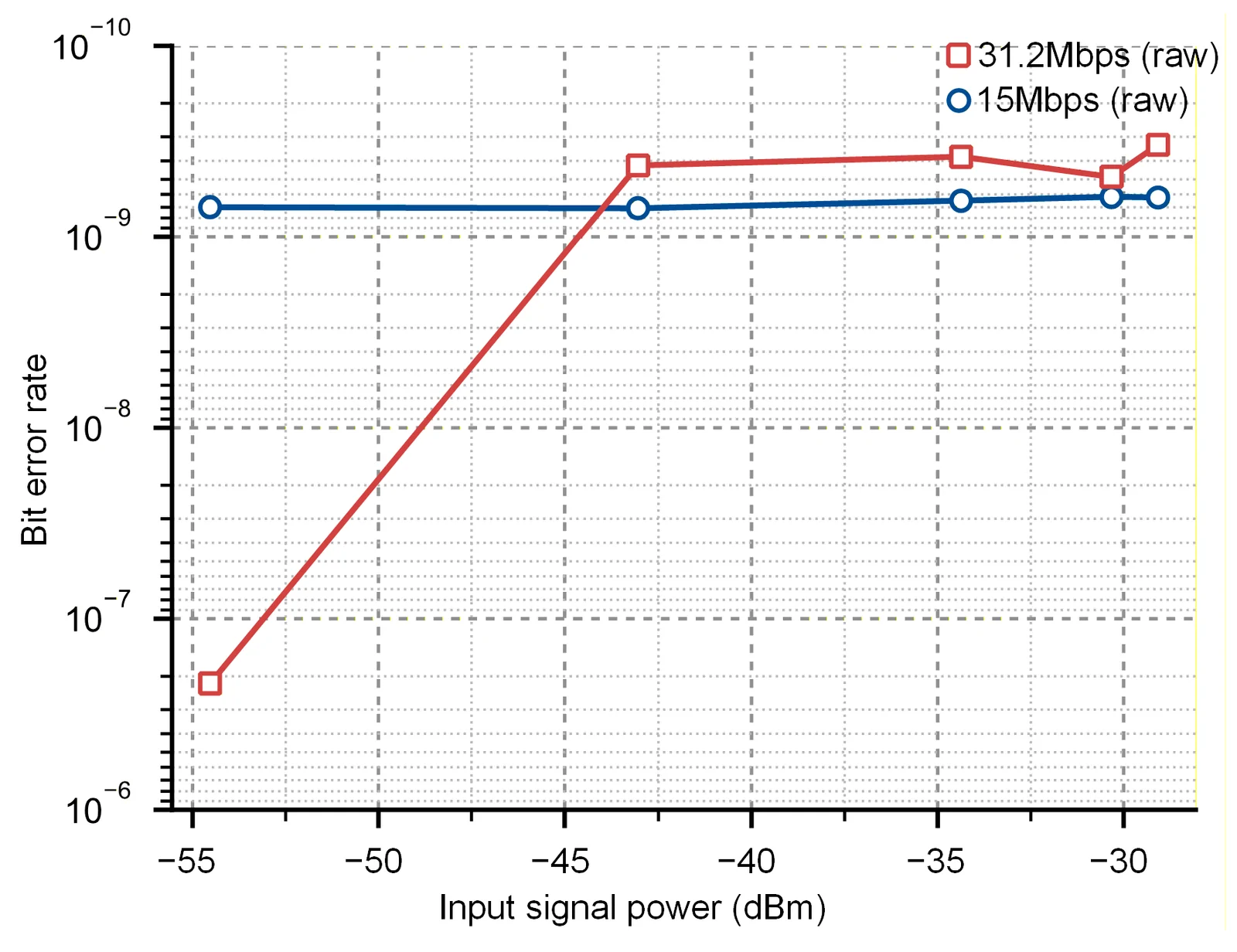
End-to-end BER as a function of calibrated receiver input power for PRBS15 inputs at raw data rates of 15 Mbps (blue; 30 Mbps Manchester-coded line rate) and 31.2 Mbps (red; 62.4 Mbps Manchester-coded line rate).

End-to-end BER was evaluated using the FPGA-based test platform shown in Figure 6b. Figure 7 presents BER as a function of calibrated receiver input power at raw input data rates of 15 and 31.2 Mbps, corresponding to Manchester-coded line rates of 30 and 62.4 Mbps, respectively. BER decreased with increasing receiver input power. At a receiver input power of −45 dBm, the measured BER values were 10^−9^ and 10^−8^ for the 15 and 31.2 Mbps raw data modes, respectively. The 31.2 Mbps raw mode matches the aggregate data stream used in the in vivo recording system. At the maximum raw input rate of 90 Mbps, corresponding to a 180 Mbps Manchester-coded line rate, the receiver maintained frame acquisition and asserted the valid-demodulation indicator. However, bit-by-bit BER at this rate was not available because the temporary fly wire return path between the receiver and FPGA limited the BER feedback bandwidth. The 90 Mbps result is therefore reported as the maximum demonstrated modulation and frame-acquisition rate, rather than as a BER-qualified operating point.

### 3.2. Performance Comparison

Table 1 compares the proposed transmitter with representative carrier-based OOK, ASK, and FSK transmitters, together with recent IR-UWB implementations. The listed designs address different operating points and therefore should not be interpreted as a single performance ranking. Recent IR-UWB transmitters achieve high pulse or data rates and compact circuit areas in advanced CMOS processes, whereas WINeRS-8 demonstrates a PLL-based 433 MHz OOK uplink in freely behaving animals. The present work targets a different design point: a PLL-less wideband OOK transmitter implemented in a mature 180 nm CMOS process, with a maximum raw input modulation rate of 90 Mbps, a tunable carrier frequency of 3.266–3.445 GHz, and a minimum measured power consumption of 26.4 mW. End-to-end BER was characterized up to 31.2 Mbps raw input, which covers the aggregate data rate used in the in vivo recording system.

### 3.3. Integration with a 128-Channel Neural Recording System

**Figure 8:**
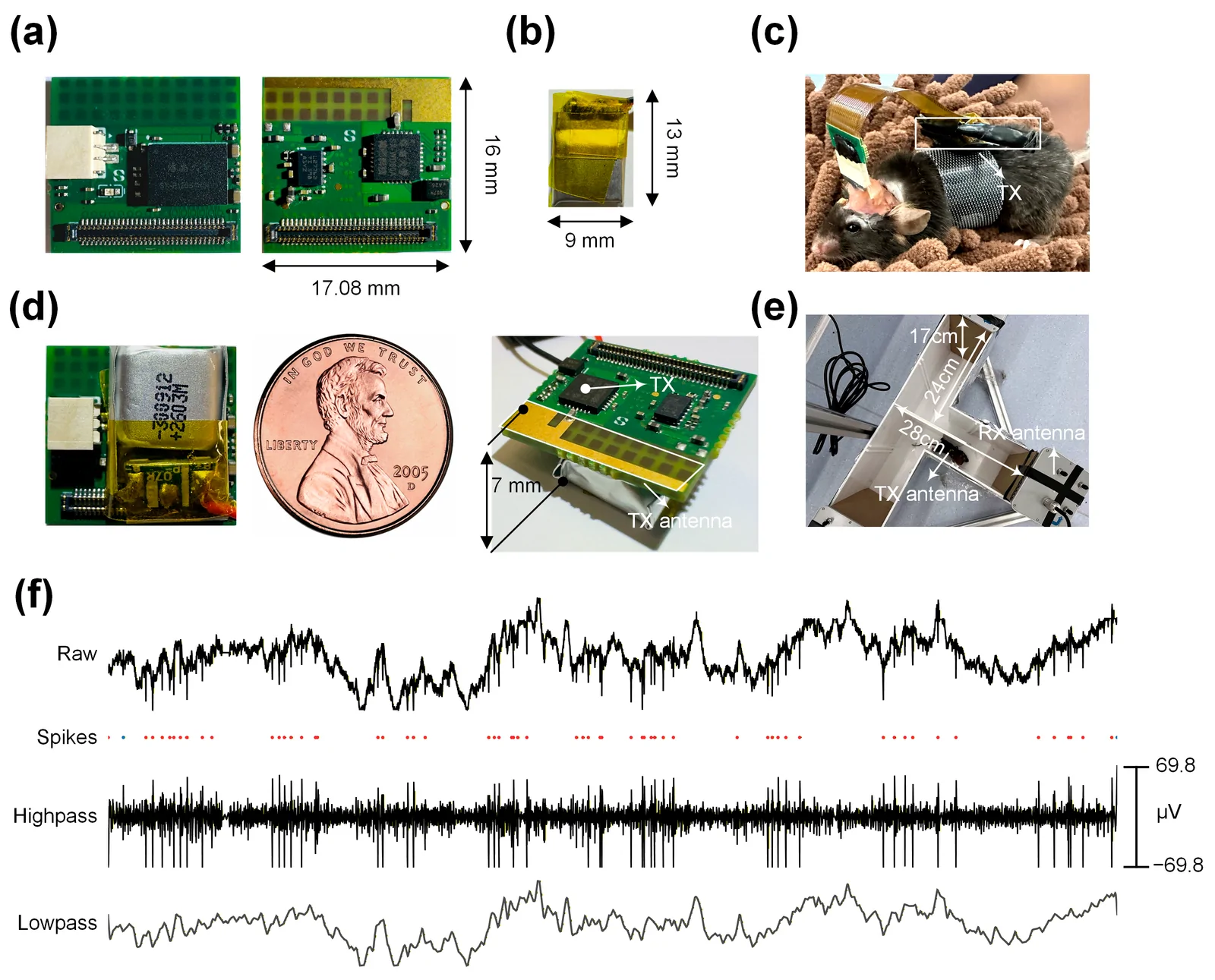
System integration and in vivo validation of the wireless neural recording device. (**a**) Integrated device combining the wireless neural-signal transmitter chip and the 128-channel neural recording chip, with a footprint of 17.08 mm × 16 mm. (**b**) 3.7 V lithium battery used for wireless operation, with a size of 13 mm × 9 mm. (**c**) The integrated device mounted on the back of the mouse and connected to the 32-channel head-mounted electrode interface; the device includes a 17 mm × 3 mm PCB-printed antenna with an approximately 6 dBi estimated gain. (**d**) Three-dimensional size comparison between the integrated device and a one-cent coin. (**e**) T-maze free exploration experiment using the 128-channel wireless neural recording device; three receiver antennas were mounted above the ends of the maze arms at a height of 17 cm, providing a transmitter–receiver distance of approximately 20–30 cm. (**f**) Representative hippocampal neural signals acquired during freely moving T-maze exploration.

Wireless neural telemetry reduces cable-induced constraints, motion artifacts, and mechanical torque during freely moving behavioral experiments. The proposed transmitter was integrated with a 128-channel neural recording chip to construct a backpack-mounted wireless acquisition device. The device included the transmitter system-on-chip (SoC), the 128-channel recording front end, a clock source, a power-management circuit, and a PCB-printed antenna. The integrated device measured 17.08 mm × 16 mm and was connected to the 32-channel head-mounted electrode interface, as shown in Figure 8a–d.

The device was powered by a 3.7 V, 40 mAh lithium-polymer battery through a 3.3 V low-dropout regulator (LDO). The transmitter consumed 26.4 mW, while the estimated total power consumption of the complete device, including the recording chip, LDO, crystal oscillator, and peripheral circuits, was approximately 70 mW. The battery has a nominal energy of 148 mWh, corresponding to an ideal operating time of approximately 2.1 h at 70 mW. After accounting for the usable battery-voltage range, LDO dropout, capacity derating, and transient current demand, the practical continuous operating time was estimated to be approximately 1.5 h.

The transmitter used a 17 mm × 3 mm PCB-printed antenna with an approximately 6 dBi estimated gain. During T-maze recording, three receiver antennas were positioned above the ends of the maze arms to cover the animal’s movement range, as shown in Figure 8e. The transmitter–receiver distance varied from approximately 20 to 30 cm. Each acquisition frame contained 128 16-bit neural-channel words and two additional 16-bit framing words. At a sampling rate of 15 kS/s, the aggregate raw data rate was therefore 31.2 Mbps, and the corresponding Manchester-coded line rate was 62.4 Mbps. Representative neural signals acquired under this operating condition are shown in Figure 8f.

### 3.4. In Vivo Wireless Neural Recording in Freely Moving Mice

To evaluate the functional usability of the proposed wireless system, wired and wireless hippocampal recordings were obtained from the same animal through the same electrode interface during the same T-maze behavioral task. The wired recording was performed first, followed by wireless recording under comparable behavioral conditions. Both systems operated at a sampling rate of 15 kS/s with 16-bit ADC resolution.

**Figure 9:**
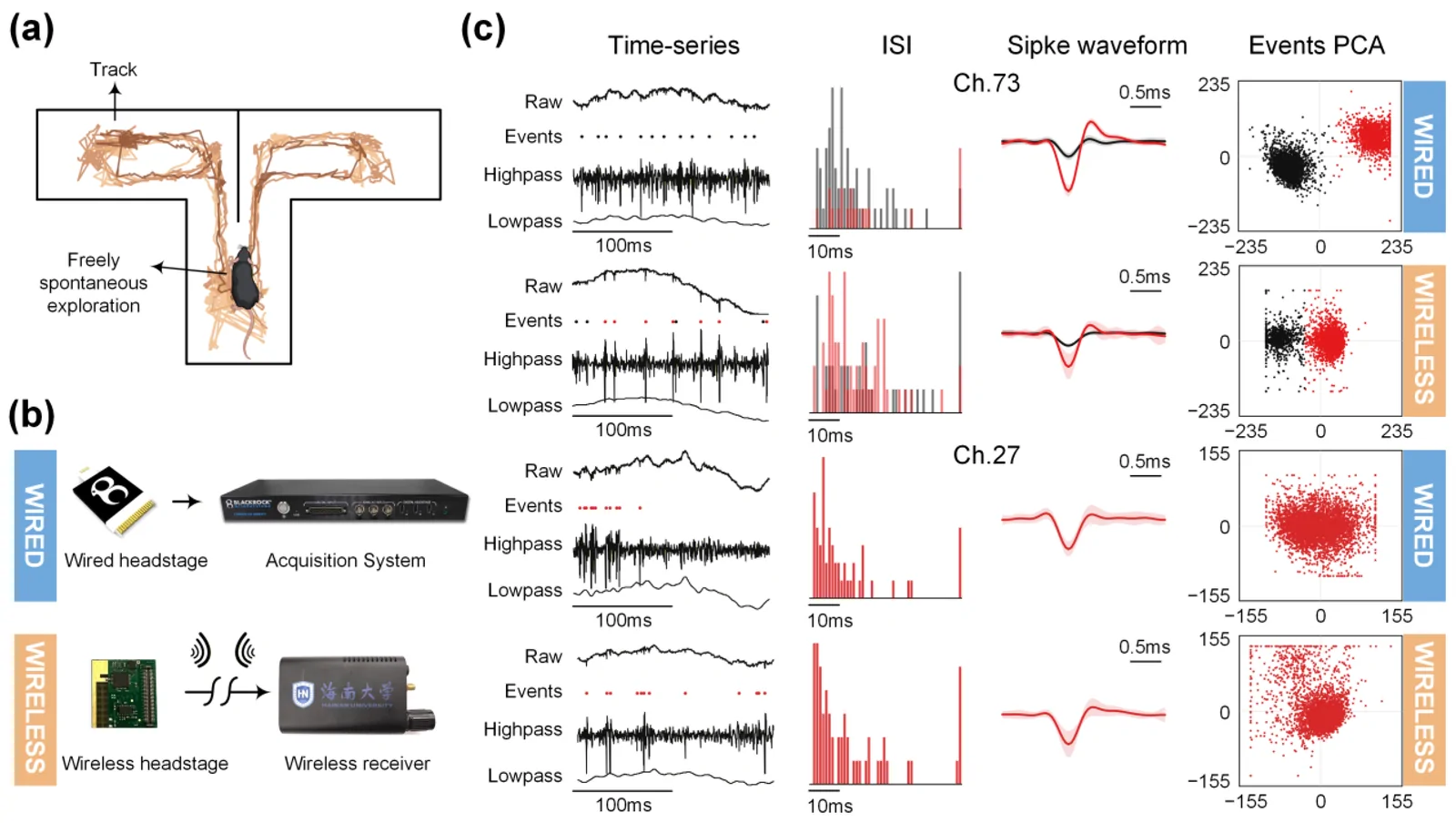
Comparison of neural signals recorded by a commercial wired system and the proposed wireless system during active T-maze exploration. (**a**) Schematic of the mouse T-maze free-exploration experiment. (**b**) Hippocampal neural signals acquired using a commercial wired recording system and the proposed wireless recording system. (**c**) Raw neural traces, LFP components, threshold-detected spike events, spike ISI, spike waveforms, and PCA projection from wired and wireless recording. In the ISI, Spike waveform, and Events PCA panels, the red and black traces and points represent two distinct event groups, respectively.

As shown in Figure 9, the wireless recordings contained identifiable raw waveforms, high-frequency spike components, and low-frequency LFP components that were also observed in the wired recordings. Threshold-based analysis of the 300–3000 Hz band-pass-filtered signals supported spike-event detection, waveform visualization, inter-spike-interval calculation, and principal-component projection. The comparison is intended as a qualitative system-level assessment of signal usability rather than a test of quantitative equivalence between the two recording systems. The detected spike events were not assigned to individual neurons and should not be interpreted as formally sorted single units.

### 3.5. Event-Locked LFP Analysis During T-Maze Exploration

**Figure 10:**
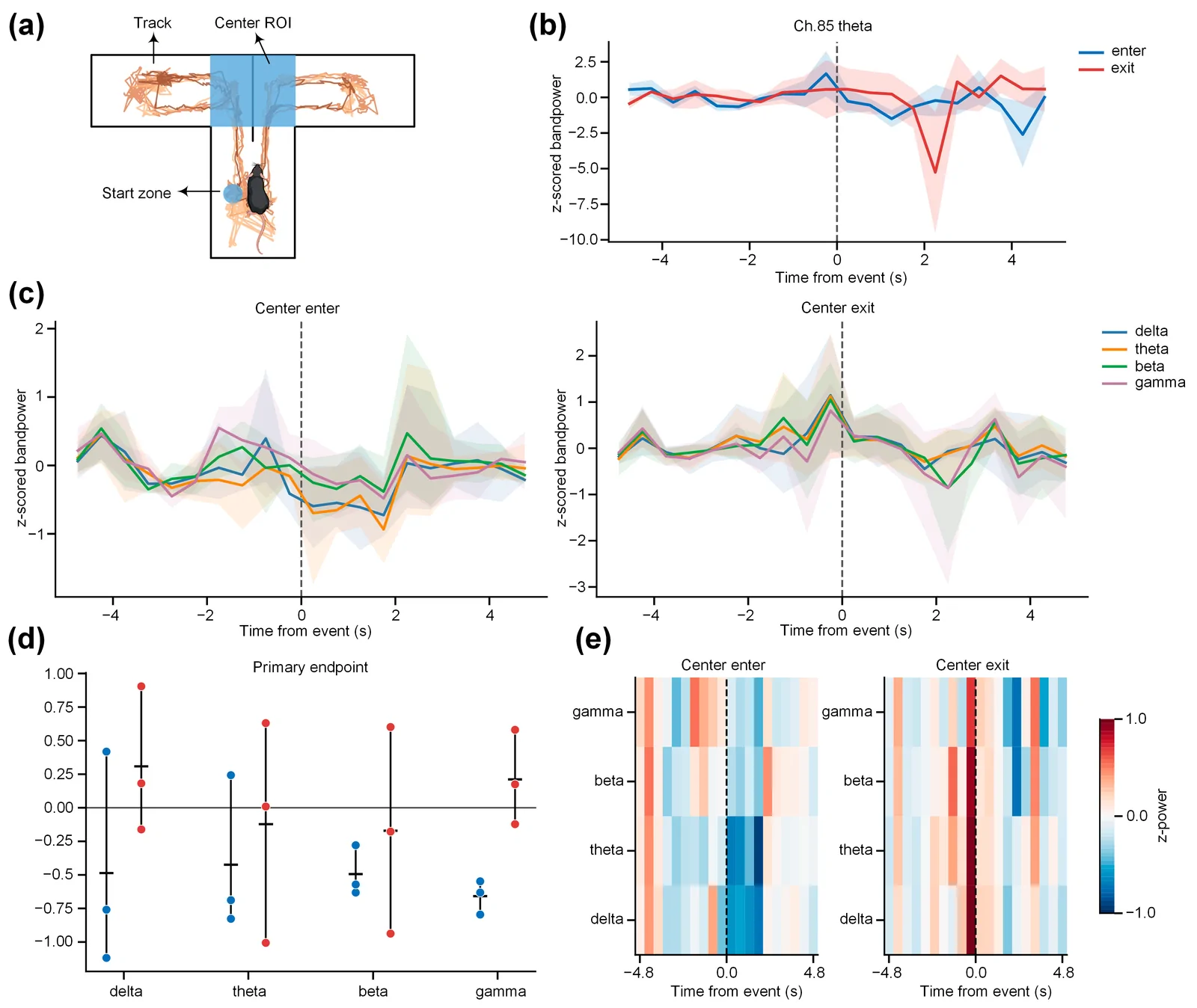
Event-locked LFP analysis during T-maze exploration. (**a**) Schematic of the mouse T-maze behavior. (**b**) Theta-band z-scored power from channel 85 around center-enter and center-exit events. (**c**) Event-locked changes in delta, theta, beta, and gamma z-scored power around center-enter and center-exit events. (**d**) Primary endpoint values in the delta, theta, beta, and gamma bands. Each colored circle represents the mouse-level mean effect estimate for an individual mouse and does not represent a confidence-interval endpoint. Blue circles indicate center-enter events, whereas red circles indicate center-exit events. (**e**) Mouse-level mean z-scored bandpower shown as heat maps.

To further examine whether the wirelessly recorded data could support behavior-related LFP analysis, an exploratory event-locked analysis was performed using T-maze free-exploration data collected from three mice over three consecutive days, as shown in Figure 10. Mouse entries into and exits from the center region were defined as center-enter and center-exit events, respectively, and were synchronized to the neural acquisition system using TTL pulses with microsecond-level timing precision. Bandpower in the delta, theta, beta, and gamma bands was log10-transformed and baseline-z-scored before event-locked analysis. The primary endpoint was defined a priori as the difference in baseline-z-scored log10 bandpower between the post-event window [0, 1 s] and the pre-event window [−2, −1 s]. The mouse was used as the highest independent statistical unit, whereas sessions, trials, and channels were treated as nested repeated observations. The results showed a decreasing trend in beta and gamma bandpower after center-enter events, whereas weaker and transient positive shifts were observed around center-exit events. This analysis indicates that the LFP signals acquired by the proposed wireless system can support event-locked bandpower analysis; however, these behavior-related changes should be interpreted as exploratory functional validation rather than as independent evidence for a specific neural mechanism.

## 4. Discussion

This work evaluates a PLL-less wideband OOK transmitter for high-throughput neural telemetry and its integration into a miniaturized wireless recording system. The architecture removes the conventional PLL-based carrier-synthesis loop and uses a free-running LC-VCO, a Gilbert-mixer isolation stage, and a current-reuse stacked PA. Schematic-level simulations of the LC-VCO showed limited frequency variation under the investigated temperature and supply conditions. Direct low-resistance loading produced a larger frequency shift, whereas the frequency was nearly unchanged for loads above 10 kΩ. The simulated phase noise was −112.6 dBc/Hz at a 1 MHz offset and −136.6 dBc/Hz at a 10 MHz offset. The transmitter accepted raw inputs up to 90 Mbps, corresponding to a 180 Mbps Manchester-coded line rate. At this maximum rate, receiver frame acquisition was observed. End-to-end BER was quantified at raw input rates of 15 and 31.2 Mbps, corresponding to encoded line rates of 30 and 62.4 Mbps. The 31.2 Mbps mode matches the aggregate data stream used in the in vivo recording system. The temporary receiver-to-FPGA return path prevented bit-by-bit BER measurement at 90 Mbps; accordingly, the maximum rate is reported as a demonstrated modulation and frame-acquisition rate rather than a BER-qualified operating point.

The comparison with prior work also places the contribution in context. WINeRS-8 used a PLL-generated 433 MHz carrier for a 9 Mbps OOK uplink within a wirelessly powered 32-channel recording and 4-channel stimulation platform [15]. The present design instead focuses on a PLL-less transmitter operating at a substantially higher raw input rate and integrated with a 128-channel recording chip. Recent IR-UWB transmitters represent another high-throughput design route and can achieve compact implementations in advanced CMOS processes [16,20]. Table 1 therefore summarizes different architectural trade-offs rather than establishing a direct ranking among systems with different modulation formats, process nodes, and system boundaries. The integrated device transmitted a 31.2 Mbps raw data stream during the in vivo experiments, corresponding to a 62.4 Mbps Manchester-coded line rate. Three receiving antennas were positioned around the T-maze to cover the animal’s movement range. The estimated total device power was approximately 70 mW, and the practical continuous operating time with the 3.7 V, 40 mAh battery was approximately 1.5 h. The wired–wireless comparison showed that the wireless data retained signal components suitable for threshold-based spike-event analysis and event-locked LFP analysis. These results support system-level neural recording, but they do not establish quantitative equivalence with the wired system or formally sorted single-unit activity.

This study has several limitations. The present VCO characterization is limited to schematic-level simulations of the standalone oscillator. Transmitter-level PVT analysis, the effects of package and antenna loading, and experimental phase-noise characterization were beyond the scope of the present study and warrant further investigation. BER at the maximum raw input data rate of 90 Mbps, comprehensive characterization of the receiver bandwidth and sensitivity, and systematic radiated-range measurements were also not included. In the present animal experiments, only the electrode recording sites were implanted, whereas the electrode interface, adapter, battery, and wireless recording device remained outside the body, with the device mounted on the mouse’s back. Therefore, the present study does not evaluate hermetic packaging, tissue heating, or the long-term reliability of a fully implanted transmitter. Future development of a fully implantable system will require thermal characterization, chronic packaging validation, long-term stability testing, and biocompatibility assessment. Scaling to multiple recording nodes will require channel spacing that accounts for free-running VCO variation, sufficient receiver bandwidth, interference management, synchronization, and data aggregation. Future validation will first use multiple transmitters and the multichannel receiver under controlled bench-top conditions, followed by simultaneous operation in a behavioral arena.

## 5. Conclusions

This work presents a low-power, PLL-less, wideband OOK neural-signal transmitter implemented in a 180 nm CMOS process. The transmitter incorporates a free-running LC-VCO, a Gilbert-mixer isolation stage, and a current-reuse stacked PA, and consumes 26.4 mW from a 3.3 V supply. It supports raw input data rates of up to 90 Mbps, corresponding to a Manchester-coded line rate of 180 Mbps, and provides a tunable carrier-frequency range of 3.266–3.445 GHz. End-to-end BER was characterized at raw input data rates of 15 and 31.2 Mbps, corresponding to encoded line rates of 30 and 62.4 Mbps, respectively; the latter covers the aggregate data stream used during the in vivo recordings. Schematic-level simulations of the LC-VCO quantified its frequency behavior under temperature, supply-voltage, and load variations, as well as its phase-noise performance. The transmitter was integrated with a 128-channel neural-recording chip and used to acquire hippocampal spike and LFP signals from freely moving mice during T-maze exploration. These results demonstrate the functional feasibility of the proposed transmitter for untethered neural recording. Future work will address BER characterization at the maximum data rate, experimental characterization of transmitter frequency stability and phase noise, radiated-link characterization, chronic packaging and thermal-safety assessment, and simultaneous multi-node operation.

## Figures and Tables

**Table 1:** Performance summary and comparison.

Ref.	Tech. (nm)	Area (mm^2^)	Fre. (GHz)	Data Rate (Mbps)	Power (mW)	Modulation
[13]	130	NA	0.915	1.5	3.7~6.6	FSK/OOK
[25]	65	NA	2.4/3.2	54	2.37/2.61	OOK
[10]	180	0.00578	0.01356	1	0.03	ASK
[11]	65	NA	0.57	0.5	<2	ASK
[23]	130	NA	0.915	8	3.66	BPSK/OOK
[15]	130	12	0.434	9	NA	OOK
[16]	28	NA	6~9	1660	9.69	4PPM + 8PSK + 4PAM impulse
[20]	28	0.043	4.8	0.2	0.38	OOK
This work	180	2	3.266~3.445	90 ^1^	26.4 (min.) ^2^	Wideband OOK

^1^ The data rate of this work refers to the raw input data rate before Manchester encoding; the corresponding Manchester-coded line rate is 180 Mbps. End-to-end BER was characterized up to a raw input rate of 31.2 Mbps, corresponding to a 62.4 Mbps encoded line rate. ^2^ denotes the minimum measured DC power consumption of this work under a 3.3 V supply.

## Data Availability

Processed data supporting the main findings of this study and the key analysis scripts are available from the corresponding author upon reasonable request. The raw in vivo neural recording data are not publicly released because of their large file size and institutional data-management requirements related to animal-experiment records and unpublished chip-system documentation. Where necessary for verification or reproducibility of the reported results, de-identified processed data or additional supporting information may be provided in accordance with institutional ethics and data-management policies.

## Abbreviations

The following abbreviations are used in this manuscript:

ADC	Analog-to-digital converter
ASIC	Application-specific integrated circuit
ASK	Amplitude-shift keying
BER	Bit error rate
BG	bandgap reference
BLE	Bluetooth Low Energy
CMOS	Complementary metal–oxide–semiconductor
DC	Direct current
FDMA	Frequency-division multiple access
FSK	Frequency-shift keying
GFSK	Gaussian frequency-shift keying
IEEE	Institute of Electrical and Electronics Engineers
IR-UWB	Impulse-radio ultra-wideband
ISI	Inter-spike interval
LC-VCO	LC voltage-controlled oscillator
LFP	Local field potential
LO	Local oscillator
OOK	On–off keying
OFDM	Orthogonal frequency-division multiplexing
PA	Power amplifier
PCB	Printed circuit board
PCA	Principal component analysis
PLL	Phase-locked loop
PRBS	Pseudorandom binary sequence
QAM	Quadrature amplitude modulation
QFN	Quad flat no-lead
QPSK	Quadrature phase-shift keying
RF	Radio frequency
SoC	System-on-chip
TTL	Transistor–transistor logic
VCO	Voltage-controlled oscillator
